# Incidence of phrenic nerve injury during pulmonary vein isolation using different cryoballoons: data from a large prospective ablation registry

**DOI:** 10.1093/europace/euae092

**Published:** 2024-04-08

**Authors:** Shinichi Tachibana, Shinsuke Miyazaki, Junichi Nitta, Yasuhiro Shirai, Yasutoshi Nagata, Yuichiro Sagawa, Yukio Sekiguchi, Yukihiro Inamura, Takeshi Sasaki, Yasuteru Yamauchi, Osamu Inaba, Yuichi Ono, Makoto Suzuki, Atsushi Suzuki, Shinsuke Iwai, Hiroyuki Okada, Akira Mizukami, Koji Azegami, Hitoshi Hachiya, Keita Handa, Kentaro Goto, Takuro Nishimura, Kenzo Hirao, Atsushi Takahashi, Tetsuo Sasano

**Affiliations:** Department of Cardiology, Japanese Red Cross Saitama Hospital, Saitama, Japan; Department of Cardiovascular Medicine, Tokyo Medical and Dental University, Yushima 1-5-45, Bunkyo-ku, Tokyo 113-8510, Japan; Department of Cardiology, Sakakibara Heart Institute, Tokyo, Japan; Department of Cardiology, Disaster Medical Center, Tokyo, Japan; Department of Cardiology, Japanese Red Cross Musashino Hospital, Tokyo, Japan; Department of Cardiology, Japanese Red Cross Yokohama City Bay Hospital, Kanagawa, Japan; Department of Cardiology, Sakakibara Heart Institute, Tokyo, Japan; Department of Cardiology, Japanese Red Cross Saitama Hospital, Saitama, Japan; Department of Cardiology, Disaster Medical Center, Tokyo, Japan; Department of Cardiology, Japanese Red Cross Yokohama City Bay Hospital, Kanagawa, Japan; Department of Cardiology, Japanese Red Cross Saitama Hospital, Saitama, Japan; Department of Cardiology, Ome Municipal General Hospital, Tokyo, Japan; Department of Cardiology, Yokohama Minami Kyosai Hospital, Kanagawa, Japan; Heart Center, Tokyo Yamate Medical Center, Tokyo, Japan; Department of Cardiology, Hiratsuka Kyosai Hospital, Kanagawa, Japan; Department of Cardiology, Soka Municipal Hospital, Saitama, Japan; Department of Cardiology, Kameda Medical Center, Chiba, Japan; Department of Cardiology, Shin-Yurigaoka General Hospital, Kanagawa, Japan; Cardiovascular Center, Tsuchiura Kyodo Hospital, Ibaraki, Japan; Division of Cardiology, Kashiwa City Hospital, Chiba, Japan; Department of Cardiovascular Medicine, Tokyo Medical and Dental University, Yushima 1-5-45, Bunkyo-ku, Tokyo 113-8510, Japan; Department of Cardiovascular Medicine, Tokyo Medical and Dental University, Yushima 1-5-45, Bunkyo-ku, Tokyo 113-8510, Japan; Arrhythmia Advanced Therapy Center, AOI Universal Hospital, Kanagawa, Japan; Department of Cardiology, Yokosuka Kyosai Hospital, Kanagawa, Japan; Department of Cardiovascular Medicine, Tokyo Medical and Dental University, Yushima 1-5-45, Bunkyo-ku, Tokyo 113-8510, Japan

**Keywords:** Cryoballoon, Catheter ablation, Atrial fibrillation, Phrenic nerve injury

## Abstract

**Aims:**

Phrenic nerve injury (PNI) is the most common complication during cryoballoon ablation. Currently, two cryoballoon systems are available, yet the difference is unclear. We sought to compare the acute procedural efficacy and safety of the two cryoballoons.

**Methods:**

This prospective observational study consisted of 2,555 consecutive atrial fibrillation (AF) patients undergoing pulmonary vein isolation (PVI) using either conventional (Arctic Front Advance) (AFA-CB) or novel cryoballoons (POLARx) (POLARx-CB) at 19 centers between January 2022 and October 2023.

**Results:**

Among 2,555 patients (68.8 ± 10.9 years, 1,740 men, paroxysmal AF[PAF] 1,670 patients), PVIs were performed by the AFA-CB and POLARx-CB in 1,358 and 1,197 patients, respectively. Touch-up ablation was required in 299(11.7%) patients. The touch-up rate was significantly lower for POLARx-CB than AFA-CB (9.5% vs. 13.6%, p = 0.002), especially for right inferior PVs (RIPVs). The touch-up rate was significantly lower for PAF than non-PAF (8.8% vs. 17.2%, *P* < 0.001) and was similar between the two cryoballoons in non-PAF patients. Right PNI occurred in 64(2.5%) patients and 22(0.9%) were symptomatic. It occurred during the right superior PV (RSPV) ablation in 39(1.5%) patients. The incidence was significantly higher for POLARx-CB than AFA-CB (3.8% vs. 1.3%, *P* < 0.001) as was the incidence of symptomatic PNI (1.7% vs. 0.1%, *P* < 0.001). The difference was significant during RSPV (2.5% vs. 0.7%, *P* < 0.001) but not RIPV ablation. The PNI recovered more quickly for the AFA-CB than POLARx-CB.

**Conclusions:**

Our study demonstrated a significantly higher incidence of right PNI and lower touch-up rate for the POLARx-CB than AFA-CB in the real-world clinical practice.

**What’s new?**

- The incidence of right PNI during the CB-PVI was significantly higher in the POLARx-CB group than AFA-CB group, and the results were consistent for both the PAF and non-PAF patients.
- Touch-up ablation was significantly more often required in the AFA-CB group than POLARx-CB group, and the difference was significant in the PAF patients and was notable for the RIPVs.
- PNI recovered more quickly in the AFA-CB group than POLARx-CB group, and the difference in the proportion of recovery at 3-month post-procedure was significant.
- The touch-up rate was significantly higher in non-PAF patients than PAF patients.

Cryoballoon (CB) ablation has become an established treatment strategy for atrial fibrillation (AF). In addition to the conventional CB [Arctic Front Advance (AFA), Medtronic],^1^ a novel CB (POLARx, Boston Scientific) (POLARx-CB) with the same 28 mm balloon size has become available.^2,3^ Despite different balloon characteristics, overall, most published data have presently shown a similar acute procedural efficacy and safety.^4–9^ However, that was retrospective, observational, and limited to high-volume centres’ experience or relatively small populations to assess complications with a low incidence. In this study, we compared the prevalence of phrenic nerve injury (PNI) during pulmonary vein isolation (PVI) between the two different CBs by analysing multi-centre prospective registry data.

This study consisted of 2555 consecutive AF patients who underwent initial PVIs using the AFA-CB or POLARx-CB (not POLARx-FIT) between January 2022 and October 2023. The data were extracted from the TMDU ablation registry (UMIN 000047063), in which all cases who underwent catheter ablation at 20 Japanese centres had been prospectively registered. The need for patient consent was waived due to the anonymized nature of the study. The study was approved by each hospital’s institutional review board. The study complied with the Declaration of Helsinki.

Anticoagulation management was according to the latest guidelines.^1^ A spiral mapping catheter (Achieve or POLARMAP) was used to advance the 28 mm CB via a steerable sheath (FlexCath Advance or POLARSHEATH). Following confirming the vein occlusion using contrast, 180–240 s freezes were applied (no bonus application). The dose was adjusted according to the time-to-isolation. The diaphragmatic electromyogram was monitored during right PV ablation (with diaphragmatic movement sensors in POLARx-CB group). If PNI was suspected (generally a >30% amplitude reduction), the freezing was terminated with active deflation. The minimal cut-off temperature was set at −60°C for AFA-CB and −70°C for POLARx-CB. The oesophageal temperature was monitored according to the operators’ preference (cut-off: 15–20°C). For PVs that could not be isolated with CBs, touch-up ablation was performed with an irrigated-tip catheter. Additional ablation beyond PVI was performed according to the operators’ discretion.

A total of 2555 patients [68.8 ± 10.9 years, 815 females, 1670 paroxysmal AF (PAF), 86 left common PV (LCPV)] were included. The PVI was performed by AFA-CB and POLARx-CB in 1358 and 1197 patients, respectively. Additional ablation beyond CB-PVI was added in 1545 (60.5%) patients. The total procedure and fluoroscopic times were 117.1 ± 57.6 and 32.0 ± 21.6 min, respectively. Procedure-related complications were identified in 146 (5.7%) patients.

Touch-up ablation was required in 299 (11.7%) patients: for the left superior, left inferior (LIPVs), right superior (RSPVs), right inferior (RIPVs), and LCPVs in 76 (3.0%), 51 (2.0%), 101 (4.0%), 151 (5.9%), and 9 (10.5%) patients, respectively. The touch-up rate was significantly lower for POLARx-CB than AFA-CB group (9.5% vs. 13.6%, *P* = 0.002). The difference was significant for RIPVs (3.1% vs. 8.4%, *P* < 0.001) (*Figure 1*). It was significantly higher for non-PAF than PAF (17.2% vs. 8.8%, *P* < 0.001). In PAF patients, it was significantly lower with POLARx-CB than AFA-CB (5.4% vs. 11.7%, *P* < 0.001), and the difference was significant for LIPVs (0.9% vs. 2.3%, *P* = 0.037) and RIPVs (1.4% vs. 7.2%, *P* < 0.001). In non-PAF patients, it was comparable between POLARx-CB and AFA-CB groups (17.0% vs. 17.4%, *P* = 0.954), except for significantly lower rate of RIPVs for POLARx-CB than AFA-CB (6.1% vs. 10.6%, *P* = 0.023).

**Figure 1:**
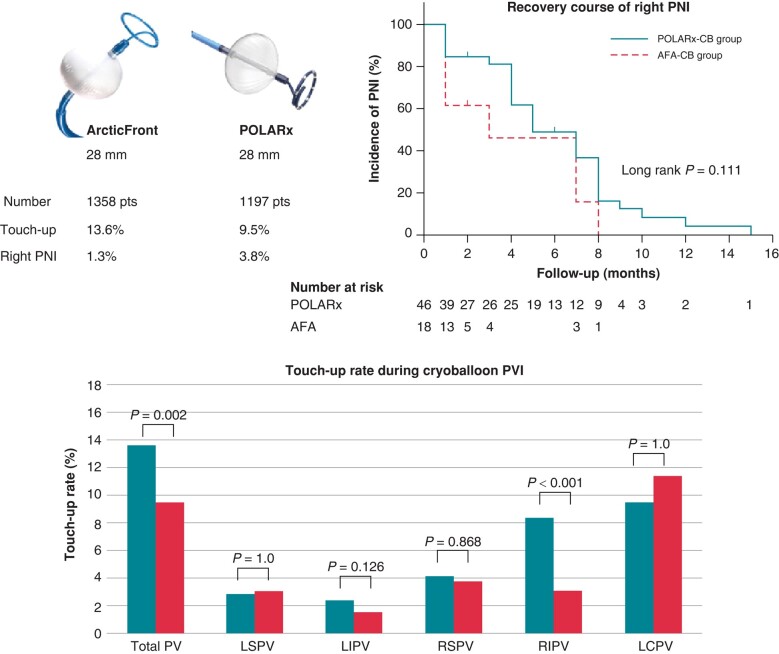
Touch-up rate and right PNI during cryoballoon pulmonary vein isolation. The touch-up rate was higher for AFA-CB than POLARx-CB, and the difference was significant for the RIPVs. The incidence of right PNI was significantly higher for POLARx than AFA-CB. Phrenic nerve injury recovered more quickly with AFA than POLARx; however, the difference was not statistically significant. LCPV, left common PV; LI(S)PV, left inferior (superior) PV; PNI, phrenic nerve injury; pts, patients; PV(I), pulmonary vein (isolation); RI(S)PV, right inferior (superior) PV.

Right PNI occurred during CB-PVIs in 64 (2.5%) patients, and 22 (0.9%) were symptomatic. It occurred during RSPV and RIPV ablation in 39 (1.5%) and 22 (0.9%) patients, respectively. The incidence was similar between PAF and non-PAF patients (2.4% vs. 2.7%, *P* = 0.723). However, the incidence was significantly higher for POLARx-CB than AFA-CB (3.8% vs. 1.3%, *P* < 0.001). The results were consistent for PAF (*P* = 0.025) and non-PAF (*P* < 0.001) (*Table 1*). A multivariate analysis revealed that POLARx-CB use and a younger age [hazard ratio 0.97, 95% confidence interval (CI) 0.95–0.99, *P* = 0.006] were associated with a higher incidence of PNI (hazard ratio 2.88, 95% CI 1.66–5.00, *P* < 0.001).

**Table 1:** Patient characteristics and incidence of right PNI

	Overall	AFA-CB	POLARx-CB	*P* value
	*n* = 2555	*n* = 1358	*n* = 1197	
Age, years	68.8 ± 10.9	69.4 ± 10.9	68.2 ± 10.8	0.005
Female gender, *n* (%)	815 (31.9%)	428 (31.5%)	387 (32.3%)	0.691
Body mass index, kg/m^2^	24.1 ± 4.0	24.3 ± 4.0	23.9 ± 3.9	0.013
Paroxysmal AF, *n* (%)	1670 (65.4%)	897 (66.1%)	773 (64.6%)	0.459
Structural heart disease, *n* (%)	322 (12.6%)	168 (12.4%)	154 (12.9%)	0.752
CHADS_2_ score	1.2 ± 1.1	1.3 ± 1.1	1.2 ± 1.1	0.01
CHA_2_DS_2_-VASc score	2.3 ± 1.5	2.3 ± 1.5	2.2 ± 1.5	0.012
Left atrial diameter, mm	38.9 ± 6.6	39.1 ± 6.3	38.6 ± 6.9	0.047
Left ventricular ejection fraction, %	61.8 ± 10.9	62.6 ± 10.9	60.9 ± 10.7	<0.001
Overall group, *n* = 2555				
Phrenic nerve injury, *n* (%)	64 (2.5%)	18 (1.3%)	46 (3.8%)	<0.001
RSPV, *n* (%)	39 (1.5%)	9 (0.7%)	30 (2.5%)	<0.001
RIPV, *n* (%)	22 (0.9%)	9 (0.7%)	13 (1.1%)	0.394
Unknown, *n* (%)	3 (0.1%)	0 (0%)	3 (0.3%)	0.205
Symptomatic, *n* (%)	22 (0.9%)	2 (0.1%)	20 (1.7%)	<0.001
PAF group, *n* = 1670				
Phrenic nerve injury, *n* (%)	40 (2.4%)	14 (1.6%)	26 (3.4%)	0.025
RSPV, *n* (%)	24 (1.4%)	7 (0.4%)	17 (2.2%)	0.041
RIPV, *n* (%)	15 (0.9%)	7 (0.4%)	8 (1.0%)	0.796
Unknown, *n* (%)	1 (0.1%)	0 (0%)	1 (0.1%)	0.941
Symptomatic, *n* (%)	12 (0.7%)	2 (0.2%)	10 (1.3%)	0.022
Non-PAF group, *n* = 885				
Phrenic nerve injury, *n* (%)	24 (2.7%)	4 (0.9%)	20 (4.7%)	<0.001
RSPV, *n* (%)	15 (1.7%)	2 (0.4%)	13 (3.1%)	0.006
RIPV, *n* (%)	7 (0.8%)	2 (0.4%)	5 (1.2%)	0.384
Unknown, *n* (%)	2 (0.2%)	0 (0%)	2 (0.5%)	0.443
Symptomatic, *n* (%)	10 (1.1%)	0 (0%)	10 (2.4%)	0.003

Values are reported as the mean ± standard deviation or number of patients (%), unless otherwise noted. The *P* values indicate the comparison between the AFA-CB and POLARx-CB groups.

AF, atrial fibrillation; *n*, number; PAF, paroxysmal atrial fibrillation; RI(S)PV, right inferior (superior) pulmonary vein.

Right PNI recovered on chest X-ray during the follow-up period in 50/64 (78.1%). It recovered within 1 month in 24/61 (39.3%) patients [15/44 (34.1%) in POLARx-group vs. 9/17 (52.9%) in AFA-group, *P* = 0.177] and within 3 months in 26/55 (47.3%) patients [16/42 (38.1%) in POLARx-group vs. 10/13 (76.9%) in AFA-group, *P* = 0.024]. Phrenic nerve injury recovered more quickly in AFA-group than POLARx-group; however, the difference was not statistically significant (*Figure 1*; *P* = 0.111).

We found that (i) the incidence of PNI was significantly higher for POLARx-CB, which was consistent for both PAF and non-PAF patients; (ii) the touch-up rate was significantly higher for AFA-CB, and the difference was significant in PAF patients and notable for RIPVs; and (iii) the touch-up rate was significantly higher in non-PAF than PAF patients. The strength of our data was the sufficiently large population to assess the complications, latest data sets, multi-centre data, and real-world data including all consecutive patients undergoing CB ablation, and all procedures were performed under diaphragmatic electromyogram monitoring.

The reported incidence of PNI during CB ablation varied (0–15%).^4–8,10–13^ This may reflect the definition of PNI, real-world experience vs. clinical trials, operator experience, and inclusion of atypical anatomies. In the real-world data, the touch-up rate per patient is 4–9% for AFA-CB,^10^ and the POLAR ICE study reported 3.2% per PV.^12^ The possible reasons for the relatively high touch-up rate in this study might be that the combined use of radiofrequency catheters and three-dimensional mapping systems was allowed by the Japanese insurance system, and therefore, the operators did not stick to the CB alone. We assumed that the lower risk of a balloon position shift owing to the lack of pop-out during the initial freezing phase increased the sheath deflection and the more compliant balloon likely decreased the need for touch-up ablation with POLARx-CB, especially for RIPVs. Conversely, the higher compliance owing to the lower inner balloon pressure might result in a wider tissue–balloon contact area, leading to aggressive cooling for the right phrenic nerve.

The limitations of this study were (i) there was no set dosing protocol because this study captured real-world data, (ii) the impact of the operators’ learning curve on the results (POLARx-CB was approved in 2021), and (iii) only acute procedural data owing to the latest dataset was available. The ongoing multi-centre randomized clinical trials (COMPARE CRYO^14^ and CONTRAST AF^15^) comparing the two CBs might provide further information.

## Data Availability

Data underlying the findings of this article are available upon reasonable request to the corresponding author.
